# Protective effects of avenanthramide-C against cisplatin-induced cardiotoxicity in rats by attenuating oxidative stress, inflammatory cytokines, and modulating p62–Keap1–Nrf2 pathway

**DOI:** 10.3389/fphar.2025.1694060

**Published:** 2025-10-08

**Authors:** Maha Abdulrahman Aldubayan

**Affiliations:** Department of Pharmacology and Toxicology, College of Pharmacy, Qassim University, Buraydah, Saudi Arabia

**Keywords:** avenanthramide-C, cisplatin, oxidative stress biomarkers, inflammatory biomarkers, cardiotoxicity

## Abstract

**Introduction:**

Cisplatin (CIS) is widely recognized as a potent antineoplastic agent, especially effective for treating various solid tumors. Nevertheless, the pathological response it induces, alongside oxidative stress and inflammation from upstream reactions, causes varying degrees of damage to multiple organs in the human body. The primary adverse effects of CIS include nephrotoxicity, neurotoxicity, ototoxicity, and gastrointestinal toxicity. CIS-induced cardiotoxicity is rare, and its prevalence remains unknown. Avenanthramide-C (AVN-C), an antioxidant compound found solely in oats (Avena sativa L.), is recognized for its significant ability to neutralize free radicals; however, the mechanism by which it exerts other protective influences remains unclear. Research indicates that AVN-C significantly reduces the expression of gene transcripts responsible for encoding pro-inflammatory cytokines when exposed to H2O2 or tumor necrosis factor-α (TNF-α). This study investigated the potential protective role of the antioxidant and anti-inflammatory properties of AVN-C in mitigating CIS-induced cardiotoxicity in rat cardiac tissue.

**Methods:**

Forty male Wistar rats were randomly assigned to 4 groups, each comprising an equal number of animals (10 animals per group), as follows: control (5%DMSO/Saline), CIS (CIS, 10 mg/kg), AVN-C (20 mg/kg), and CIS + AVN-C groups. Blood plasma was collected from the retro-orbital plexus for the evaluation of biochemical parameters, including lactate dehydrogenase (LDH), creatine kinase (CK-MB), and troponin I. Cardiac tissues were extracted to evaluate oxidative stress markers, including reactive oxygen species (ROS), malondialdehyde (MDA), and superoxide dismutase (SOD). Additionally, inflammatory markers such as TNF-α, interleukin (IL)-1β, IL-6, and nuclear factor kappa B (NF-κB) were assessed. The heart tissues were also examined for the protein and mRNA expressions for p62, Kelch-like ECH-associated protein 1 (Keap1), and nuclear factor erythroid 2-related factor 2 (Nrf2).

**Results:**

The CIS group exhibited significantly increased LDH, CK-MB, troponin I, MDA, ROS, TNF-α, IL-6, IL-1β, NF-κB, and Keap1 levels. However, AVN-C administration led to a significant reduction in these marker levels. Additionally, CIS + AVN-C treatment resulted in significantly increased p62, Nrf2, and SOD levels compared to the CIS group.

**Conclusion:**

AVN-C may protect against CIS-induced cardiotoxicity by reducing oxidative stress and inflammation, possibly activating the p62-Keap1-Nrf2 pathway. Histopathologically, heart tissues treated with CIS + AVN-C were less damaged than tissues treated with the CIS group. These findings suggest AVN-C as a promising therapeutic agent against CIS-induced cardiotoxicity. Nonetheless, the absence of echocardiographic assessments remains a key limitation, and future studies incorporating these evaluations are warranted to strengthen translational relevance.

## Graphical Abstract

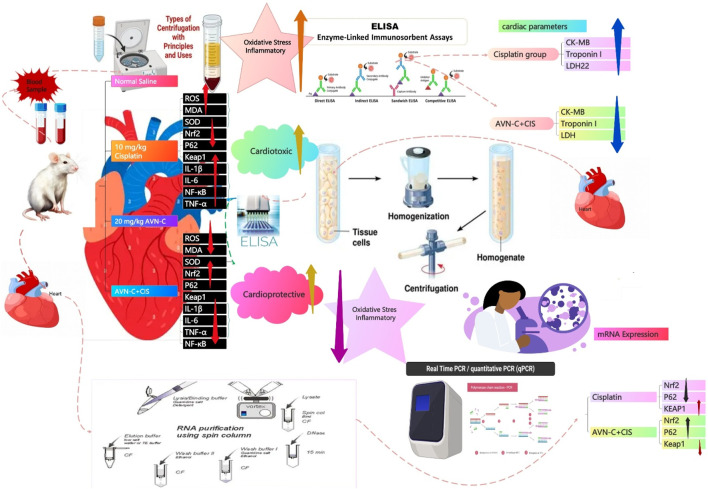

## 1 Introduction

Cisplatin (CIS) is a widely used chemotherapeutic agent for the treatment of tumors (Alhowail, 2025; Alotayk et al., 2023). Despite its efficacy, the therapeutic application of CIS is constrained by significant adverse effects, notably its tendency to damage normal tissues, resulting in hepatotoxicity, nephrotoxicity, and cardiotoxicity (Alhowail, 2025; Alotayk et al., 2023).

Cardiotoxicity serves as a dose-limiting factor that profoundly impacts the clinical outcomes of chemotherapy (Bhutani et al., 2025). There is a growing body of evidence suggesting that the primary mechanisms underlying chemotherapy-induced cardiotoxicity are oxidative stress and inflammation as well as mitochondrial damage and calcium flux alteration (Abudalo et al., 2024). The cardiotoxicity linked to CIS, along with its other adverse side effects, presents a significant challenge in cancer treatment, often necessitating the premature discontinuation of its use by many patients (Rachma et al., 2024). This interruption frequently prevents patients from fully benefiting from cancer treatment, underscoring the urgent need for therapeutic approaches that ensure both safety and cardioprotection. While the mechanisms underlying the antitumor activity of CIS are moderately well elucidated, the molecular pathways responsible for its cardiotoxicity remain inadequately understood (Rachma et al., 2024; Xia et al., 2022).

Alkylating agents, including CIS, were reported to have observed cardiotoxicity in 6%–30% of patients (Shil et al., 2025). Cardiac damage may be associated with CIS through its potential to elevate the levels of biomarkers such as creatine kinase (CK-MB), lactate dehydrogenase (LDH), and troponin I (Abudalo et al., 2024). CIS-induced cardiotoxicity is a well-established phenomenon, marked by notable changes in electrocardiographic readings and the emergence of various arrhythmias (Kapoor et al., 2023). These include atrial fibrillation, supraventricular tachycardia, ventricular arrhythmias, and occasional sinus bradycardia (Kapoor et al., 2023; Raja et al., 2021). Cardiotoxic effects have the potential to lead to the development of congestive heart failure and may result in sudden cardiac death (Shil et al., 2025).

Oxidative stress, characterized by an imbalance between the formation of reactive oxygen species (ROS) and the body’s antioxidant mechanisms, is a key contributor to CIS-induced cardiac damage (Yildirim et al., 2022). This oxidative imbalance disrupts multiple cellular pathways, particularly those involved in inflammation and necrobiosis (Huang et al., 2021). Elevated ROS levels can stimulate nuclear transcription factors, which subsequently enhance the release of pro-inflammatory cytokines, thereby amplifying inflammatory responses (Ju et al., 2024; Soliman et al., 2018). Nuclear factor erythroid 2-related factor 2 (Nrf2) plays a critical role in protecting cells from oxidative stress and damage (Tan et al., 2021). The stabilization and activation of Nrf2, facilitated by the sequestration of Kelch-like ECH-associated protein 1 (Keap1) through p62, a primary regulator responsible for Nrf2 degradation, are essential for enhancing cellular defenses against oxidative stress (Tan et al., 2021). Furthermore, oxidative stress is directly associated with elevated malondialdehyde (MDA) levels and heart failure progression (Abudalo et al., 2024). A previous study investigating the isolated hearts of rats treated with CIS revealed reduced coronary flow and increased cardiac enzyme leakage, which were correlated with elevated ROS levels and lipid peroxidation (Xia et al., 2022).

An inflammatory response is an inevitable consequence, often occurring as a secondary event following cellular or tissue damage due to toxic exposure (Bender et al., 2025). Emerging research indicates that CIS triggers the production of a range of inflammatory cytokines and chemokines, and enhances the movement of the redox-sensitive transcription factor nuclear factor kappa B (NF-κB) from the cytosol into the nucleus (Zaaba et al., 2025). This translocation triggers the synthesis of tumor necrosis factor-alpha (TNF-α), a pro-inflammatory cytokine that is crucially involved in the inflammation induced by CIS in kidney tubular cells and cardiomyocytes (Yildirim et al., 2022; Zaaba et al., 2025). CIS has been reported to markedly increase the production of myocardial TNF-α and elevate myocardial myeloperoxidase activity in both rats and mice (Yildirim et al., 2022). Although there is limited documentation on CIS-induced cardiac inflammation, it is conceivable that the myocardial inflammation observed following CIS treatment may result from molecular mechanisms similar to those identified in the kidney (Dugbartey et al., 2016). In summary, CIS triggers oxidative stress and activates pro-inflammatory mediators, which in turn enhance oxidation and inflammation, ultimately leading to cellular damage associated with cardiac toxicity. Current clinical interventions to counteract CIS-induced cardiotoxicity have shown limited efficacy (Hesari et al., 2024). Accordingly, formulating effective therapeutic interventions is urgently required to mitigate CIS-related cardiotoxic effects.

Recently, oat grain (*Avena sativa* L.) has become a focal point of academic research due to its recognized health benefits (Alemayehu et al., 2023). Originating from oat grain, avenanthramides (AVNs) are nitrogen-containing phenolic compounds commonly present in human food that are characterized by their low molecular weight and recognized for their substantial antioxidant and anti-inflammatory properties (Pretorius and Dubery, 2023). AVNs have been associated with a lower risk of colon cancer by influencing apoptosis, a mechanism that inhibits cellular proliferation (Fu et al., 2019). Furthermore, AVNs can mitigate CIS-induced nephrotoxic alterations and are considered renoprotective agents during CIS administration (Amir et al., 2019).

Oats contain AVNs in various forms, among which AVN-C is the most dominant and possesses the most potent antioxidant effect (Xie et al., 2024). AVN-C plays a significant role in mitigating H_2_O_2_-induced oxidative stress by lowering intracellular free radical levels and reducing the expression of antioxidant gene transcripts (Wang and Eskiw, 2019; Xie et al., 2024). Additionally, it contributes to a decline in the expression of gene transcripts for pro-inflammatory cytokines following exposure to H_2_O_2_ or TNF-α (Wang and Eskiw, 2019). Furthermore, cellular AVN-C administration suppresses TNF-α (Amir et al., 2019). The application of oat extract effectively suppresses the secretion of pro-inflammatory cytokines (interleukin [IL]-6) and chemokines (IL-8) in endothelial cells when stimulated by IL-1β (de Bruijn et al., 2019). Previous studies have established the potential of AVN-C to alleviate inflammation and oxidative stress in human skin fibroblasts (Wang and Eskiw, 2019), as well as its protective effect on critical organs, including the liver, lungs, and kidneys, from various types of damage (Alwaili et al., 2024; Amir et al., 2019). Thus, AVN-C offers a promising approach for mitigating the detrimental effects of CIS on cardiac health (Alwaili et al., 2024; Amir et al., 2019). However, the role of AVN-C in modulating the cardiotoxicity induced by CIS has not been systematically evaluated.

Cisplatin’s nephrotoxic and neurotoxic effects have been the subject of extensive research, yet its cardiotoxic effects have not been as thoroughly examined (Hu et al., 2018; Rachma et al., 2025). As a result, protective measures have predominantly targeted the kidneys and nervous system, leaving the heart as a relatively neglected area of study (Rachma et al., 2025). Simultaneously, AVN-C, a compound recognized for its antioxidant and anti-inflammatory capabilities (Alwaili et al., 2024), has been extensively explored in terms of neuroprotection (Jin et al., 2020) and vascular health (Park et al., 2021), but its potential role in mitigating chemotherapy-induced cardiac injury has not been investigated. This disconnect between the study of cisplatin’s cardiotoxicity and the potential therapeutic benefits of AVN-C reveals a significant research gap: the cardioprotective potential of AVN-C against cisplatin-induced cardiac damage remains uninvestigated.

This study aimed to evaluate the cardioprotective properties of AVN-C against CIS-induced toxicity in male rats, focusing on alterations in myocardial biomarker activities, including oxidative stress, pro-inflammatory cytokines, and regulating of activation of the p62–Keap1–Nrf2 signaling pathway, which are associated with the increasing incidence of cardiotoxicity among patients with cancer undergoing CIS-based chemotherapy. At present, no known therapeutic drug ameliorates CIS-induced cardiac damage. The results of this study may help identify a novel natural treatment to alleviate the adverse effects of CIS-induced cardiotoxicity.

## 2 Materials and methods

### 2.1 Drugs

CIS at 1 mg/mL was sourced from EBEWE Pharma Ges. mbH, Nfg. KG (Unterach am Attersee, Austria). AVN-C methyl ester (CAS number 955382-52-2; catalog number CAY10011336-1) was obtained from Cayman Chemical (Ann Arbor, MI, United States). AVN-C was subsequently pre-dissolved in dimethyl sulfoxide (5% DMSO/Saline).

### 2.2 Animals

Forty male rats, each weighing 200–250 g, were sourced from the College of Pharmacy at Qassim University, Saudi Arabia. The rats were housed in cages with an ambient temperature of 25 °C ± 2 °C. The experimental protocols outlined in this study were approved by the Animal Care and Use Committee at the Deanship for Scientific Research, Qassim University (reference number 23-67-07). All procedures were conducted in strict compliance with the ethical standards established by Qassim University.

### 2.3 Survival rate and body weight

The daily assessment of survival rates offered vital insights into the study’s progression. Animals that had died were swiftly removed from the study environment. Frequent evaluation of body weight provides crucial insights into overarching health patterns. Systematic assessments conducted every 3 days enable the detection of subtle variations and contribute to the early identification of potential health concerns.

### 2.4 Experimental design and drug administration

In this study, animals were randomly assigned to 4 separate groups, each comprising 10 animals per group (*n* = 10). The negative control group was orally administered 5% DMSO/saline for 10 days. On day 2, the CIS group was intraperitoneally injected with a single dose of CIS at 10 mg/kg (Eisa et al., 2021). From days 1–10, the AVN-C group was administered AVN-C at 20 mg/kg via daily oral gavage (Amir et al., 2019; Li Ji et al., 2003; Xie et al., 2024). The CIS + AVN-C groups received a single intraperitoneal injection of 10 mg/kg CIS (day 2), in conjunction with daily oral administration of 20 mg/kg AVN-C over 10 days.

### 2.5 Plasma separation and enzyme-linked immunosorbent assays

Seven blood samples from each of the seven animals in each group were collected via retro-orbital puncture and placed into tubes containing heparin. Plasma was isolated by centrifuging the samples at 4,000 rpm for 10 min, after which it was transferred to new tubes. The plasma was subsequently used to indicate the cardiac injury by quantifying plasma cardiac enzymes, including CK-MB (Cat. no. RK03570; Abclonal, Woburn, MA, United States), lactate, LDH (Cat. no. A7625; ABclonal), and troponin I (Cat. no. RK04071; ABclonal), using a commercially available rat ELISA kit (ABclonal) following the manufacturer’s protocols. Absorbance measurements for each well were performed at 450 nm utilizing a Microplate Reader (BioTek Instruments, Winooski, VT, United States) (Rana and Soni, 2008).

### 2.6 Preparation of heart tissue homogenates and enzyme-linked immunosorbent assays

On day 10, the animals were evaluated for seven animals per group to ensure all the groups were equal. After the animal rats died, they were removed, and the rats were placed in a glass chamber and anesthetized with CO2 (Alsikhan et al., 2023), before being euthanized through decapitation. Then, applied through the excision of rat hearts, a 10% tissue sample was homogenized in 0.1 M potassium phosphate buffer (pH 7.4). The homogenate was then subjected to centrifugation at 1,000 × *g* for 15 min at 4 °C. The resulting supernatant was analyzed for oxidative stress biomarkers, including MDA (Cat no. RK15281; ABclonal), ROS (Cat no. RK15283; ZellBio GmbH, Lonsee, Germany), and superoxide dismutase (SOD) (Cat no. RK07054; ABclonal), as well as inflammatory biomarkers such as IL-1β (Cat no. RK00009; ABclonal), IL-6 (Cat no. RK00020; ABclonal), TNF-α (Cat no. RK00029; ABclonal), and NF-κB (Cat no. RK03838; ABclonal). Moreover, proteins involved in the signal pathway, including p62 (Cat no. MBS3809397; MyBioSource, San Diego, CA, United States), Keap1 (Cat no. RD-KEAP1-Ra; Reddot Biotech, Katy, TX, United States), and Nrf2 (Cat no. A78517; Antibodies.com, Cambridge, United Kingdom), were measured using commercially available rat ELISA kits following the manufacturer’s protocols. Absorbance for each well was measured at 450 nm using a Microplate Reader (BioTek Instruments) (Alsikhan et al., 2023).

### 2.7 Reverse transcription–quantitative PCR (RT-qPCR)

RT-qPCR is a highly sensitive and quantitative method employed to measure mRNA expression levels. In this research, the mRNA expression of Nrf2, p62, and Keap1 was analyzed. The extraction of total RNA from tissue samples was performed according to the manufacturer’s protocol, using the GET Total RNA kit (cat. no. 787-123, Biosciences, San Diego, CA, United States). The specific primers were procured from Integrated DNA Technologies (Coralville, IA, United States) (Table 1), diluted to a concentration of 10 μM/μL using double-distilled water, and subsequently stored at −20 °C. The purity of RNA in each sample was assessed using a NanoDrop ND-2000c spectrophotometer (Thermo Fisher Scientific, Labtech, United Kingdom). Reverse transcription and PCR quantification were performed utilizing the ABScript II One-Step SYBR Green RT-qPCR Kit (cat. no. RK20404, ABclonal Technology). Each sample, containing 400 ng of RNA, was reverse-transcribed into complementary DNA. Subsequently, the PCR was performed using the AriaMx Real-Time PCR System (Agilent Technologies, Santa Clara, CA, United States) according to the manufacturer’s instructions. The reaction mixture included SYBR Green RT-qPCR buffer, ABScript II enzyme mix, 10 μM of both forward and reverse primers, ROX II reference dye (50×), and total RNA. It was adjusted to a final volume of 20 µL with RNase-free water. The thermocycling conditions were as follows: reverse transcription consisted of one cycle for 5 min at 42 °C, and pre-denaturation consisted of one cycle for 1 min at 95 °C, followed by 40 cycles for 5 s at 95 °C and 32–34 s at 60 °C.

**TABLE 1:** Primer sequences for quantitative real-time PCR.

Gene	Sequence (5’_3′)	Length (bp)	References
Nrf2 FWD	CCC​ATT​GAG​GGC​TGT​GAT​CT	60	Zhao et al. (2021)
Nrf2 REV	GCC​TTC​AGT​GTG​CTT​CTG​GTT		
Keap1 FWD	GGC​TGG​GAT​GCC​TTG​TAA​AG	57	Zhao et al. (2021)
Keap1 REV	GGG​CCC​ATG​GAT​TTC​AGT​T		
P62 FWD	GGA​ACT​GAT​GGA​GTC​GGA​TAA​C	80	Luo et al. (2019)
P62 REV	GTG​GAT​GGG​TCC​ACT​TCT​TT		
GAPDH FWD	ACT​CCC​ATT​CTT​CCA​CCT​TTG	104	Alsikhan et al. (2023)
GAPDH REV	CCC​TGT​TGC​TGT​AGC​CAT​ATT		

To ensure the accuracy of the findings, each sample was analyzed in duplicate across three independent experiments. The data were processed automatically using AiraMx software, following the plate configuration for a comparative quantitation experiment. Gene expression levels were normalized against the housekeeping gene Gapdh. To assess changes in mRNA expression, the abundance of each gene’s transcript was calculated relative to the abundance of the Gapdh transcript.

### 2.8 Histopathological evaluation of cardiac tissue

Hearts tissues were excised, rinsed in ice-cold saline to remove blood, and fixed in 10% neutral buffered formalin. Then, cardiac tissue blocks were sectioned at a thickness of 4–5 µm using a rotary microtome. The sections were mounted on glass slides and stained with hematoxylin and eosin following standard protocols (Bukhari et al., 2022). Stained sections were then examined under a light microscope (Olympus) under ×100 magnification.

### 2.9 Statistical analysis

Data analysis was performed using GraphPad Prism 9 software (GraphPad Software, La Jolla, CA, United States). To evaluate oxidative stress and inflammatory biomarkers alongside cardiac functions, a one-way ANOVA was conducted, followed by the Tukey–Kramer test to facilitate multiple comparisons. A *p*-value <0.05 was deemed to indicate statistical significance.

## 3 Results

### 3.1 Effect of CIS on survival

**FIGURE 1:**
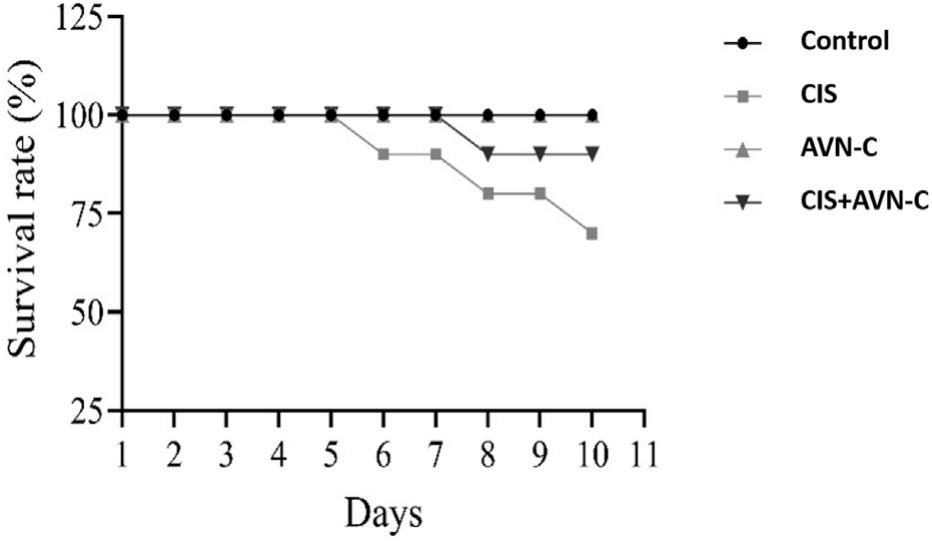
Illustrative effect of CIS and AVN-C treatments on survival rats that showed a reduction in the survival rate of rats in the CIS alone and CIS + AVN-C groups relative to the control group (n = 10).

By the 10th day, CIS treatment resulted in a 30% mortality rate among the rats. However, co-administration with AVN-C resulted in a 10% mortality rate. The control and AVN-C groups survived throughout the study duration (Figure 1).

### 3.2 Effect of CIS on bodyweight

**FIGURE 2:**
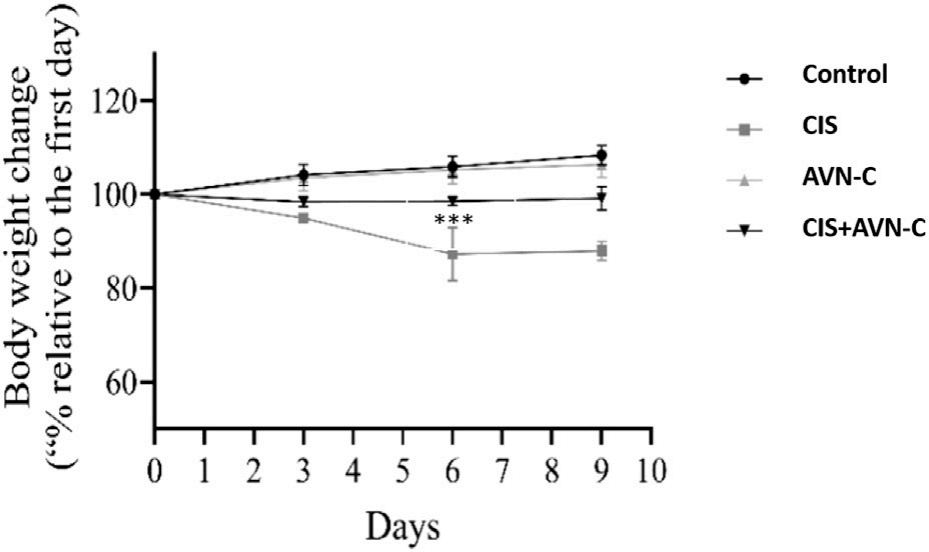
The effects of CIS and AVN-C on the body weight of rats were systematically recorded for all the groups. The data are reported as mean ± SEM (n = 7). Statistical analysis was conducted using one-way ANOVA, followed by the Tukey–Kramer *post hoc* test. Significance was assessed at levels of CIS groups ***p < 0.001, relative to the control and CIS + AVN-C.

The CIS-treated rats exhibited a significant reduction in body weight on days 6 and 9 compared to the control and the CIS + AVN-C groups (Figure 2).

### 3.3 Effects of AVN-C in conjunction with CIS on cardiac parameters

**FIGURE 3:**
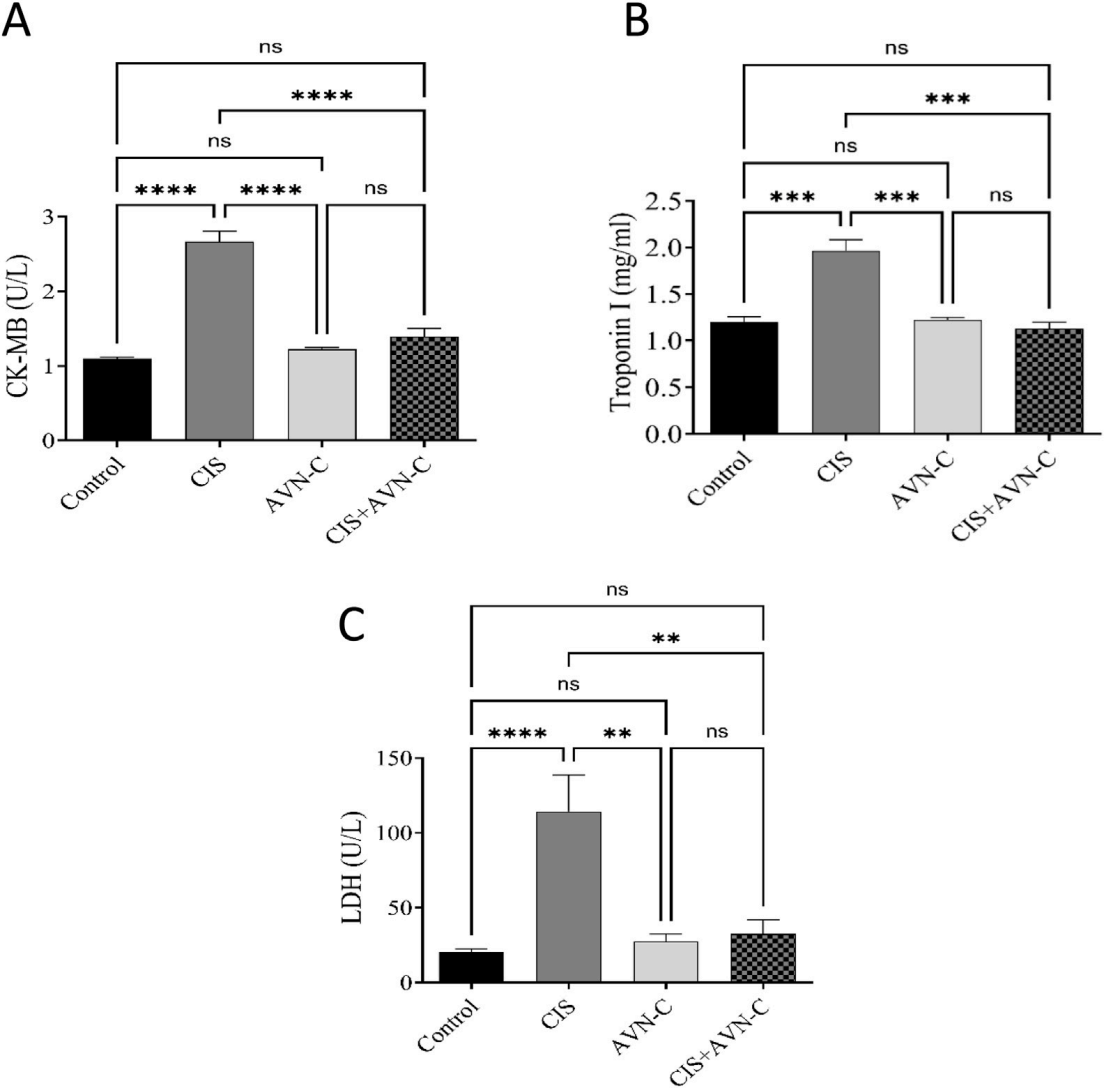
Effect of AVN-C in combination with CIS on cardiac biomarkers. **(A)** Influence of AVN-C with CIS on creatine kinase (CK-MB) activity. **(B)** Influence of AVN-C with CIS on troponin I activity. **(C)** Influence of AVN-C with CIS on lactate dehydrogenase (LDH) activity. The data are reported as mean ± SEM (n = 7). Statistical analysis was conducted using one-way ANOVA, followed by the Tukey–Kramer *post hoc* test. Significance was assessed at levels of *p < 0.05, **p < 0.01, and ****p < 0.0001, relative to the control and CIS-treated groups.

The CIS group exhibited remarkably elevated CK-MB, troponin I, and LDH levels compared with the control group (Figures 3A–C). Conversely, the CIS + AVN-C group demonstrated a significant decrease in CK-MB, troponin I, and LDH activities compared with the CIS-treated group.

### 3.4 Impact of AVN-C in conjunction with CIS on oxidative stress markers in rat heart tissue

**FIGURE 4:**
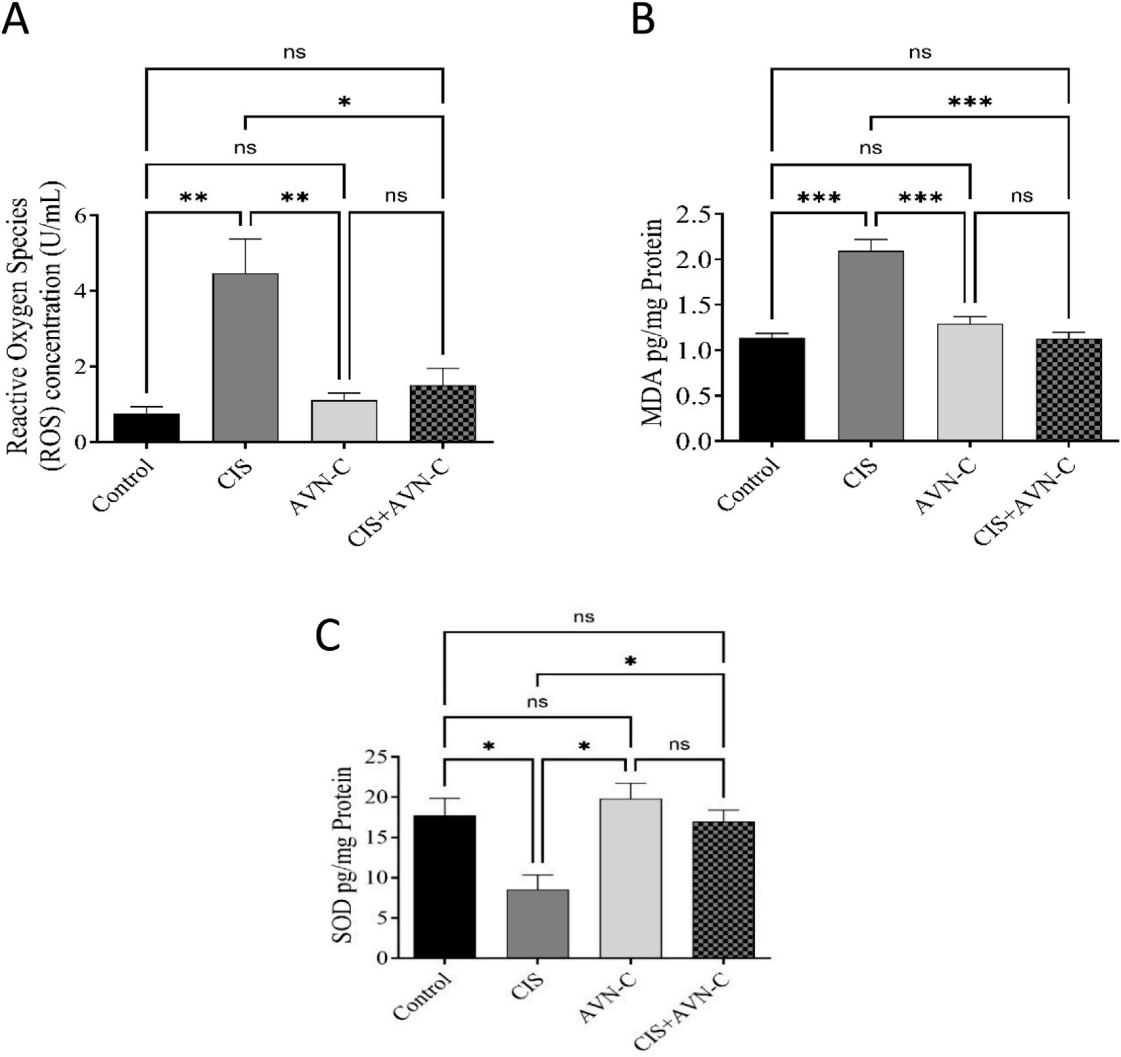
Effect of AVN-C in combination with CIS on MDA and ROS levels. **(A)** Influence of AVN-C with CIS on MDA levels. **(B)** Influence of AVN-C with CIS on ROS levels. **(C)** Influence of AVN-C with CIS on SOD activity. The data are reported as mean ± SEM (n = 7). Statistical analysis was conducted using one-way ANOVA, followed by the Tukey–Kramer *post hoc* test. Significance was assessed at levels of *p < 0.05, **p < 0.01, and ***p < 0.001, relative to the control and CIS-treated groups.

MDA and ROS levels significantly increased in the CIS-treated rats compared with those in the control and ANV-C + CIS-treated groups (Figures 4A,B). Conversely, SOD activity was significantly decreased in the CIS-treated group compared with the control and ANV-C + CIS-treated groups (Figure 4C).

### 3.5 Impact of AVN-C in conjunction with CIS on inflammatory markers in rat cardiac tissues

**FIGURE 5:**
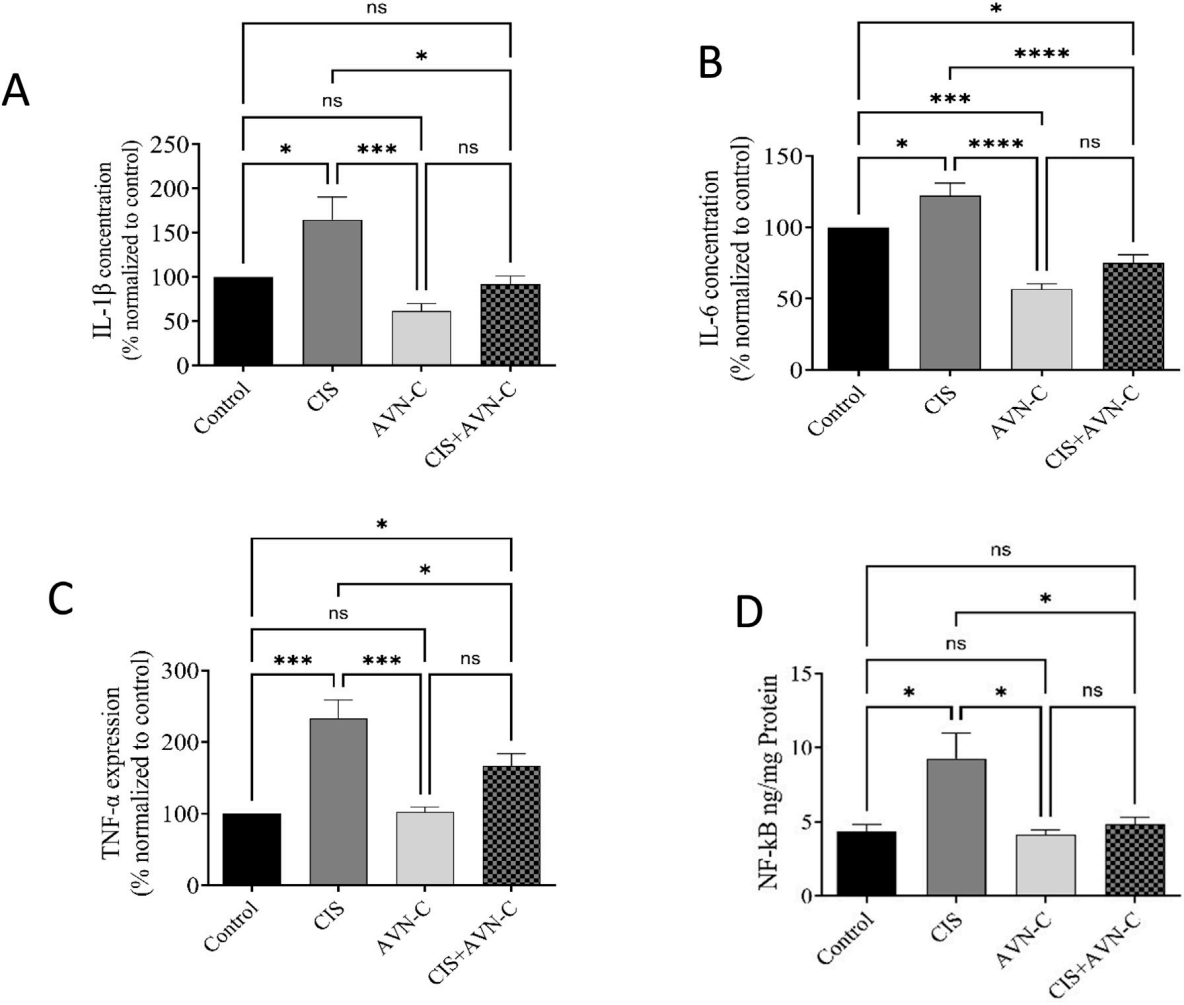
Effect of AVN-C on CIS-induced alterations in inflammatory markers. **(A)** Interleukin (IL)-1β, **(B)** IL-6, **(C)** tumor necrosis factor-alpha (TNF-α), and **(D)** nuclear factor kappa beta (NF-κB) in the cardiac tissue of rats. The data are reported as mean ± SEM (n = 7). Statistical analysis was conducted using one-way ANOVA, followed by the Tukey–Kramer *post hoc* test. Significance was assessed at levels of *p < 0.05, ***p < 0.001, and ****p < 0.0001, relative to the control and CIS-treated groups.

IL-1β, IL-6, TNF-α, and NF-κB levels significantly increased in the CIS-treated group compared with those in the control group. However, treatment with CIS + AVN-C resulted in a significant decrease in inflammatory markers associated with CIS-treated rats (Figures 5A–D).

### 3.6 Effect of AVN-C in conjunction with CIS on signaling pathway proteins in rat cardiac tissues

**FIGURE 6:**
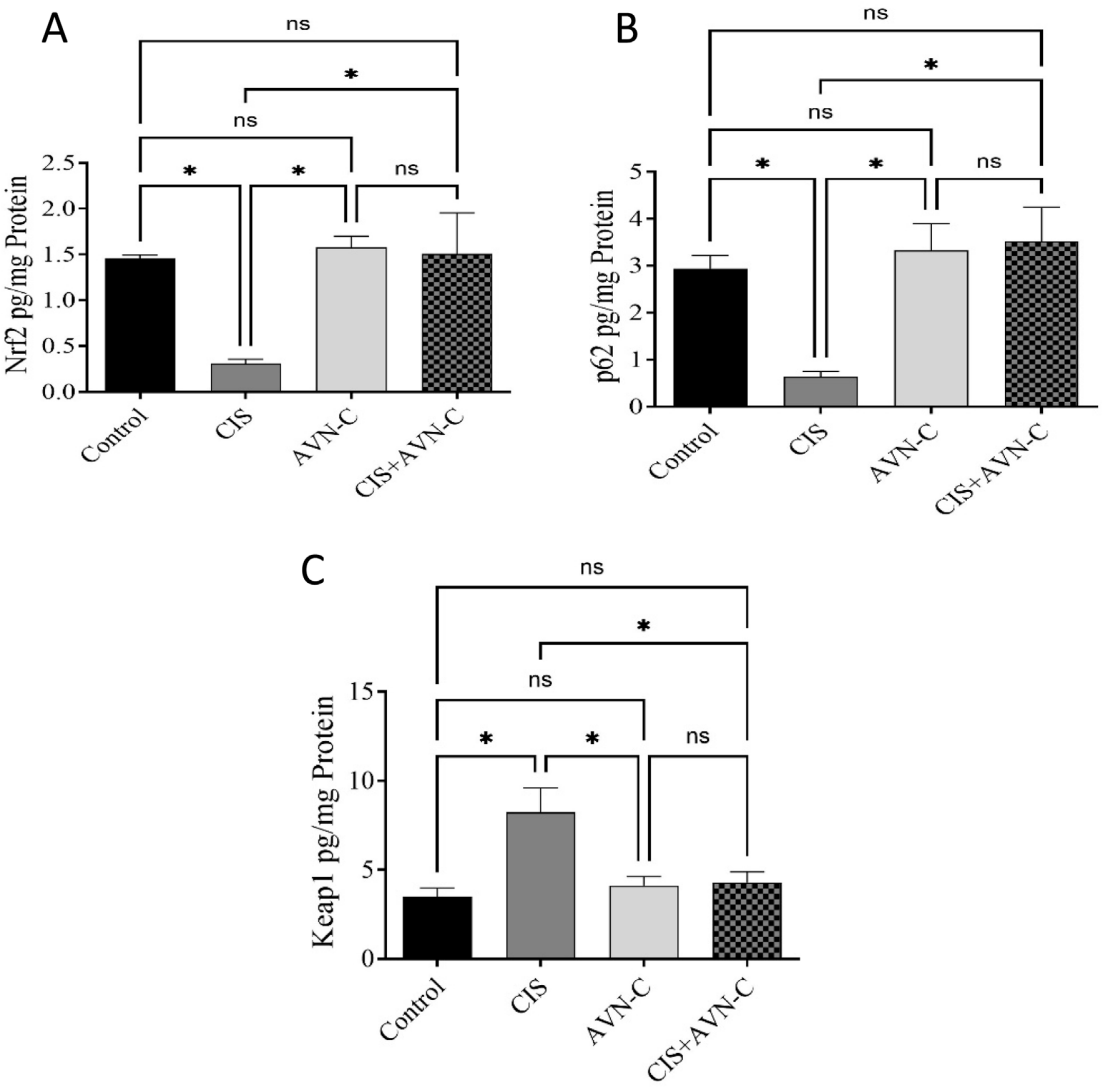
Effect of AVN-C on CIS-induced alterations to Nrf2, P62, and Keap1 levels. **(A)** Nrf2, **(B)** P62, and **(C)** Keap1 in the cardiac tissues of rats. The results are expressed as mean ± SEM (*n* = 10). The data are reported as mean ± SEM (n = 7). Statistical analysis was conducted using one-way ANOVA, followed by the Tukey–Kramer *post hoc* test. Significance was assessed at the level of *p < 0.05, relative to the control and CIS-treated groups.

In CIS-treated rats, a significant reduction in Nrf2 and p62 levels was observed. In contrast, Keap1 expression levels were significantly elevated in the CIS group compared with those in the control group. Conversely, in the AVN-C + CIS group, Nrf2 and P62 expression increased, whereas Keap1 levels decreased compared with those in the CIS group. Furthermore, no significant differences in the expression of each protein were detected between the AVN-C and control groups (Figures 6A–C).

### 3.7 Effects of CIS and AVN-C on Nrf2-Keap1-p62 pathway mRNA expression

**FIGURE 7:**
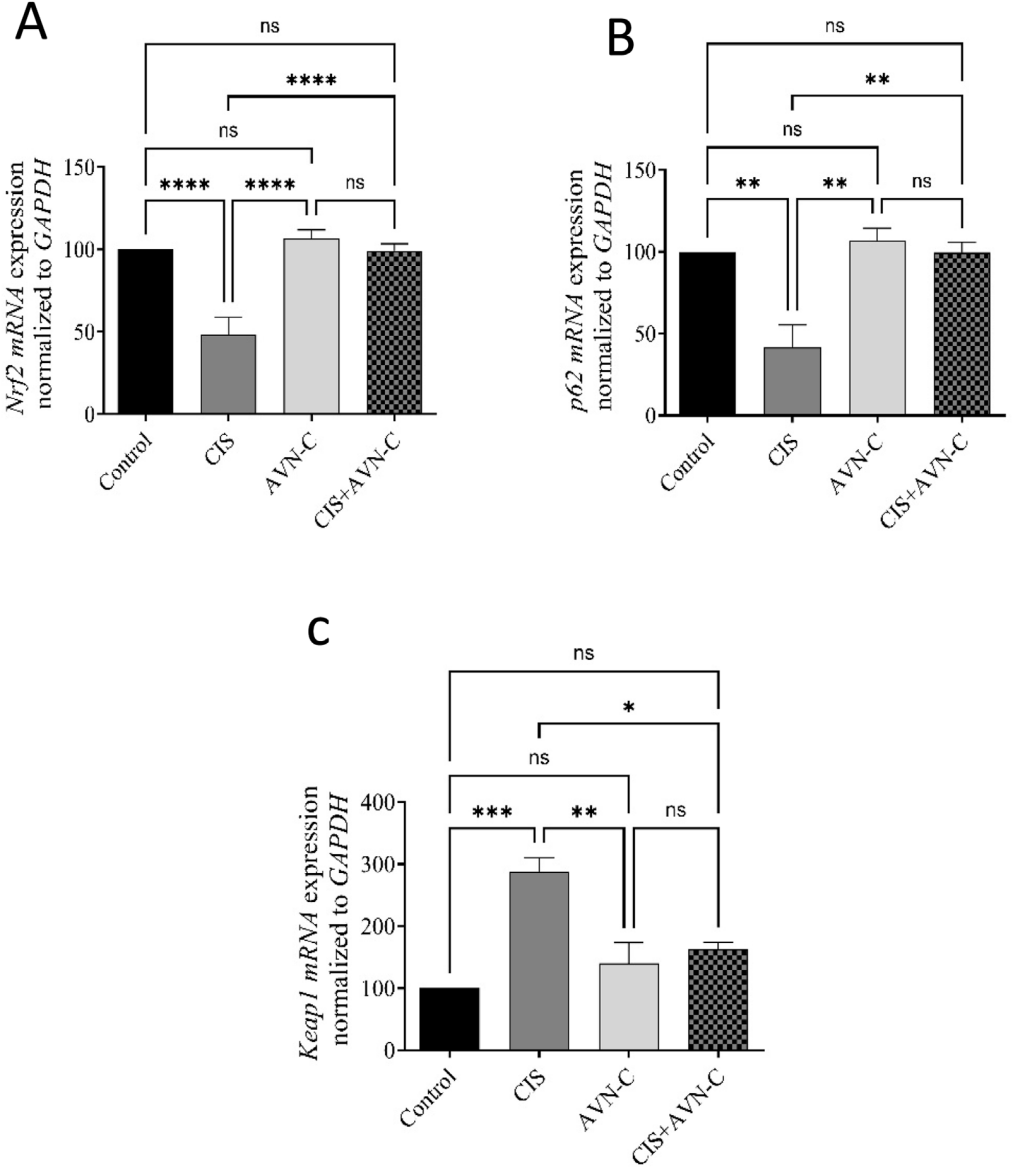
Effect of AVN-C on CIS-induced alterations to Nrf2, P62, and Keap1 levels. **(A)** Nrf2, **(B)** P62, and **(C)** Keap1 in the cardiac tissues of rats. The data are reported as mean ± SEM (n = 7). Statistical analysis was conducted using one-way ANOVA, followed by the Tukey–Kramer *post hoc* test. Significance was assessed at levels of *p < 0.05, **p < 0.01, ***p < 0.001, and ****p < 0.0001, relative to the control and CIS-treated groups.

CIS significantly decreased the mRNA expression of Nrf2 and p62. However, treatment with AVN-C reversed the changes caused by CIS in rats. In contrast, Keap1 gene expression was significantly elevated in the CIS group compared with that in the control group. In contrast, in the AVN-C + CIS group, Keap1 gene expression decreased compared to that in the CIS group (Figures 7A–C).

### 3.8 Histological staining

**FIGURE 8:**
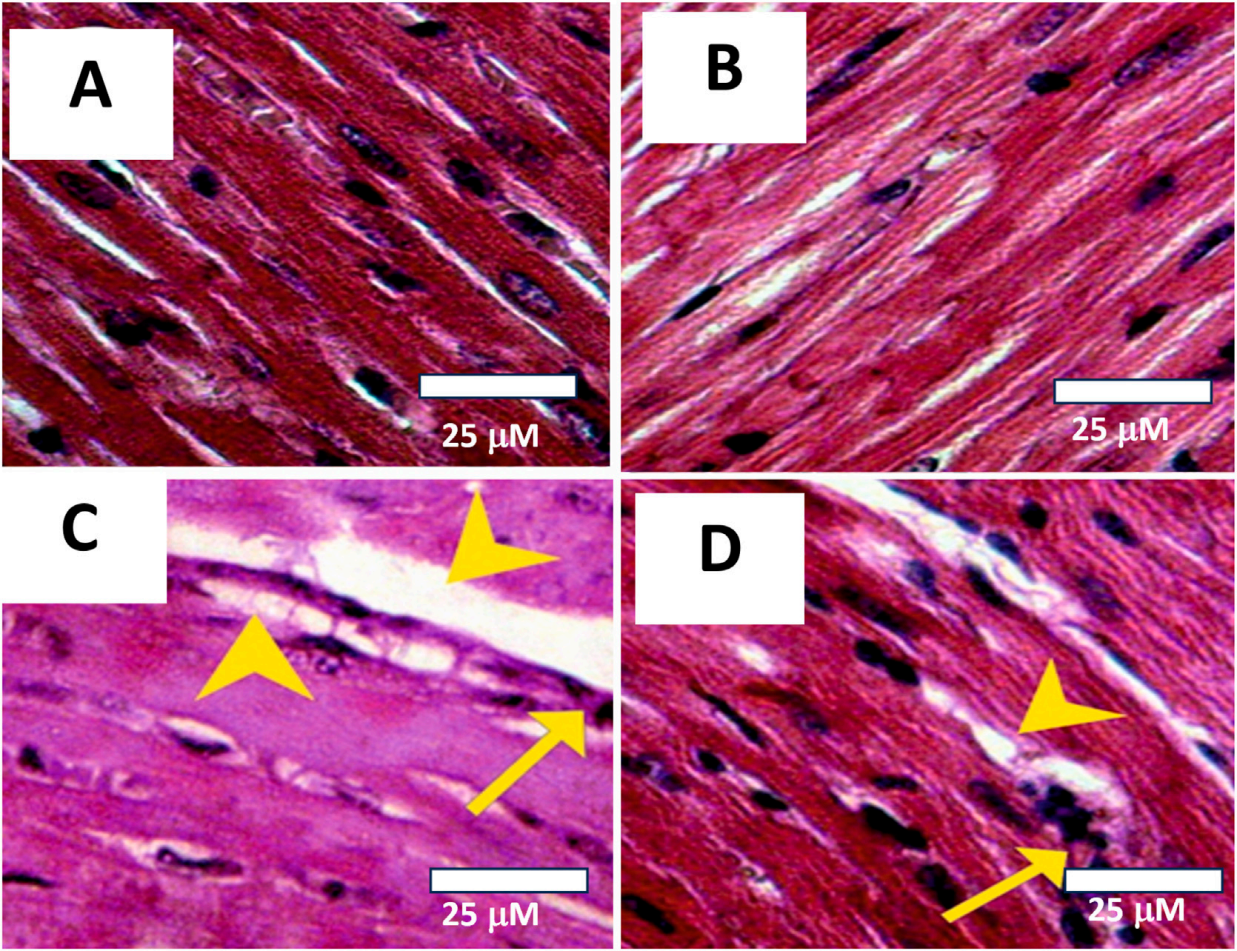
Photomicrographs of histological sections from cardiac tissues across different experimental groups. **(A)** Control group: Demonstrates normal myofibrillar structure with apparent striations. Histopathology Score: 0 (Normal). **(B)** AVN-C treated group: Shows normal to very mild myofibrillar morphology, with no signs of necrosis, inflammation, or tissue damage. Histopathology Score: 0–1 (Normal to very mild). **(C)** The group treated with CIS exhibited significant myocardial damage, as indicated by the arrowheads, which is suggestive of toxic injury. The histopathology score for this group was 3–4, denoting marked to severe damage. **(D)** In contrast, the AVN-C group associated with CIS displayed mild myocardial damage, also indicated by arrowheads, but this was less severe compared to the CIS group. The histopathology score for the AVN-C group was 2, indicating moderate damage.

Upon examination through light microscopy, heart sections from both the Control and AVN-C-treated groups demonstrated normal myofibrillar morphology. The myofibrils appeared intact, systematically organized, and aligned parallel to adjacent structures, with no evidence of necrosis, inflammation, or damage (Figures 8A,B). On the other hand, heart tissue samples from the CIS group rats revealed moderate to severe changes in myofibrillar structure, marked by significant myofiber disruption, edema, and the presence of inflammatory cell infiltration (yellow arrows). Necrosis and cellular breakdown are observable (Figure 8C). Heart sections from CIS treated with AVN-C showed mild tissue damage, including some infiltration of inflammatory cells (marked by arrows), disrupted fibers, and early stages of necrosis (marked by arrowheads) when viewed under light microscopy (Figure 8D).

## 4 Discussion

In this study, we evaluated the efficacy of AVN-C treatment in alleviating CIS-induced cardiotoxicity in rats through *in vivo* studies, including hematologic and biochemical investigations. Furthermore, we investigated the mechanisms of action of AVN-C against CIS-induced cardiotoxicity by modulating antioxidant and anti-inflammatory pathways, as well as regulating the p62–Keap1–Nrf2 signaling pathway in rats.

In this study, rats subjected to CIS treatment exhibited a significant reduction in final body weight compared to the control groups. This observation is consistent with prior research, which indicates that although rats resumed gaining weight after discontinuation of CIS treatment, their overall growth rate remained inferior to that of the control rats (Mokhtar et al., 2021). Cisplatin administration is often linked to weight loss, primarily due to its emetogenic effects, which lead to reduced appetite, gastrointestinal toxicity, and diarrhea (He et al., 2023). AVN-C administration resulted in a significant increase in body weight, which may be attributed to its anti-inflammatory and antioxidant properties.

Biochemical markers, such as cardiac troponin I, CK-MB, and LDH, play a crucial role in diagnosing cardiac injury (Aldubayan et al., 2020). CIS is recognized for its ability to compromise the integrity of cardiac cell membranes, thereby facilitating the release of intracellular proteins such as cardiac troponin I, CK-MB, and LDH (Aldubayan, 2023; Alhowail and Aldubayan, 2023; Bertinchant et al., 2003). Previous study have shown a marked increase in the concentrations of LDH, CK-MB, and troponin I relative to those in control groups following CIS administration (El-Awady et al., 2011). Our study showed that the levels of these markers increased following CIS treatment, indicating cardiac damage. However, treatment with AVN-C markedly decreased these marker levels, signifying a protective effect against the cardiac toxicity induced by CIS. Therefore, our research proposes that AVN-C holds potential as a therapeutic agent for mitigating cardiotoxicity associated with CIS. Oxidative stress is pivotal in CIS-induced cardiac damage (Afzal et al., 2023); it exacerbates ROS accumulation, triggering a lipid peroxidation cascade that compromises membrane integrity (An et al., 2024). MDA, a lipid peroxidation product, serves as an indicator of the extent of injury to biological tissues (Aldubayan et al., 2024; Cordiano et al., 2023). Elevated MDA levels can severely impair cell membranes, thereby causing cumulative oxidative stress (Cordiano et al., 2023). The present study demonstrated that CIS treatment exacerbates oxidative stress by significantly increasing ROS and MDA levels, while also reducing SOD activity. AVN-C interference, which substantially reduces ROS and MDA levels, highlights the possibility of restoring this balance, thereby protecting cardiac cells from oxidative impairment and increasing SOD activity. In line with this finding, a prior study demonstrated the ability of AVN-C to reduce ROS levels and protect synaptic ribbons from methotrexate-induced oxidative damage (Shore et al., 2022). Furthermore, our results are consistent with those of a previous study, which reported that AVN-C significantly improved the reversal of changes in hepatic antioxidant/oxidant hemostasis in doxorubicin-challenged rats owing to its ability to inhibit lipid peroxidation and prevent oxidative stress (Alwaili et al., 2024).

The interaction between oxidative stress and inflammation serves as a principal mechanism underlying CIS-induced cardiotoxicity (Olaniyan et al., 2023). Excessive ROS production can induce inflammatory responses, thereby exacerbating cardiac injury (Mittal et al., 2014). TNF-α, a pivotal inflammatory cytokine, initiates an inflammatory cascade that contributes to myocardial apoptosis and ventricular remodeling (Schumacher and Naga Prasad, 2018). Conversely, IL-1β and IL-6 can be produced in cardiac depression. Extensive research has indicated that CIS promotes the production of TNF-α, IL-1β, and IL-6 within cardiac tissue, thereby directly contributing to tissue damage (Katkenov et al., 2024). A prior study demonstrated that AVN treatment of cells significantly inhibited TNF-α and reduced IL-1β and IL-6 (Wang and Eskiw, 2019). Furthermore, recent findings have shown that AVN-C effectively reduced TNF-α levels in rats subjected to titanium dioxide nanoparticles (Amir et al., 2019). This suppression of TNF-α was associated with an improvement in CIS-induced renal injury (Amir et al., 2019). In addition, the expression of pro-inflammatory cytokines is regulated by NF-κB, a key pathogenic factor in both acute and chronic inflammation (Jung et al., 2017). These findings suggest that the ameliorative impact of AVN-C may result from the reduction in TNF-α and NF-κB, which are involved in the pathogenesis of cardiotoxicity associated with CIS. Our results demonstrate that AVN-C significantly reduced the levels of the inflammatory cytokines TNF-α, NF-κB, IL-1β, and IL-6. This dual achievement of mitigating oxidative stress and inflammation highlights the comprehensive cardioprotective mechanism of AVN-C.

Inflammatory responses can lead to increased oxidative stress (Xu et al., 2013). The Keap1–Nrf2 pathway is recognized as a key antioxidant defense mechanism that regulates oxidative stress (Chen and Maltagliati, 2018). Under normal conditions, Nrf2 is kept in an inactive state in the cytoplasm through its association with the negative regulator Keap1 (Silva-Islas and Maldonado, 2018). In response to stress, the Keap1–Nrf2 complex undergoes dissociation, which mitigates the suppressive influence of Keap1 on Nrf2. This process results in the activation of Nrf2, allowing it to migrate into the nucleus (Silva-Islas and Maldonado, 2018). Recent studies have identified the role of p62 in regulating oxidative stress through the Keap1–Nrf2 pathway. The interaction between p62 and Keap1 enhances the nuclear translocation of Nrf2, thereby initiating its downstream signaling cascade (Bartolini et al., 2018; Silva-Islas and Maldonado, 2018). The upregulation of Nrf2 resulted in increased expression of antioxidant enzymes, which led to a reduction in ROS levels (Abudalo et al., 2024). In the present study, the administration of CIS significantly reduced p62 and Nrf2 protein and mRNA expression levels while concurrently increasing Keap1 protein and mRNA expression levels, aligning with the findings of a previous study (Abudalo et al., 2024; Jia et al., 2022). Conversely, our results showed that treatment with AVN-C led to an increase in p62 and Nrf2 protein and mRNA expression levels, as well as a reduction in Keap1 protein and mRNA expression levels.

In this study, the histopathological staining results for the group treated solely with AVN-C revealed that the cardiac sections largely maintained their histological integrity, comparable to the control group. The myocardial fibers were well-aligned, exhibiting minimal or no visible edema or inflammation, and the nuclei preserved their normal position and morphology. The absence of structural damage or pathological changes suggests that AVN-C does not induce cardiotoxicity and may possess inherent cardioprotective properties. This finding is consistent with previous reports highlighting AVN-C’s antioxidant and anti-inflammatory roles (Alwaili et al., 2024; Park et al., 2021; Wang and Eskiw, 2019), which could contribute to maintaining myocardial integrity even under pharmacological exposure. In contrast, the group treated with CIS exhibited significant histopathological changes, including extensive disruption of myocardial fibers, cytoplasmic vacuolization, infiltration of inflammatory cells (as indicated by yellow arrows), and early necrotic alterations. These features are indicative of acute myocardial injury, potentially arising from oxidative stress, inflammatory processes, or cardiotoxic exposure. The pronounced severity of these lesions emphasizes the susceptibility of cardiac tissue under pathological conditions and underscores the necessity for effective cardioprotective interventions.

Notably, the group administered AVN-C following CIS therapy showed a moderate improvement in myocardial structure when compared to the group that received only CIS treatment. Although specific histological abnormalities, such as inflammatory infiltration (indicated by an arrow) and myofibrillar disorganization (indicated by an arrowhead), remained observable, the overall integrity of the tissue appeared to be partially restored. This observation suggests a potential ameliorative effect of AVN-C on myocardial damage. The observed reduction in inflammatory markers and structural deterioration suggests that AVN-C may mitigate cardiac injury through mechanisms involving anti-inflammatory effects or free radical scavenging.

This study has important strengths. A significant strength is the consistent use of strain, age group, and sex. Moreover, while numerous studies have demonstrated the cardioprotective effects of AVN-C, no prior studies have evaluated oxidative stress and inflammatory biomarkers, as well as the modulation of the p62–Keap1–Nrf2 signaling pathways in rats treated with CIS. Nonetheless, a limitation of this study is the absence of echocardiography for assessing cardiac function. Therefore, future research should incorporate echocardiography to validate and extend our findings. The present study elucidates cardiac damage induced by CIS, as evidenced by distinct biochemical and histological alterations. These alterations are characterized by elevated levels of cardiac markers, such as CK-MB, troponin I, and LDH, alongside increased ROS and MDA levels and heightened immunoreactivity of IL-1β, IL-6, TNF-α, and NF-κB. Furthermore, the AVN-C + CIS treatment resulted in a notable increase in p62, Nrf2, and SOD levels compared with those in the CIS group. A marked increase in CIS-induced Keap1 expression was also observed relative to that in the control and AVN-C treatment groups. This study substantiates the cardioprotective efficacy of AVN-C in alleviating CIS-induced cardiotoxicity. This is demonstrated by enhanced cardiac function, decreased oxidative stress and inflammatory biomarkers, and modulation of the p62–Keap1–Nrf2 signaling pathways through protein and gene expression relative to those in rats treated with CIS alone. AVN-C confers cardioprotection, both by preventing histological damage when administered alone and by reducing the severity of injury when administered with CIS.

These results suggest that AVN-C supplementation may offer a protective strategy against the cardiotoxic effects associated with CIS, but further studies including functional and clinical investigations are required.

## Data Availability

The original contributions presented in the study are included in the article/supplementary material, further inquiries can be directed to the corresponding author.
